# The complete chloroplast genome of *Cenchrus ciliaris* (Poaceae)

**DOI:** 10.1080/23802359.2018.1481795

**Published:** 2018-06-11

**Authors:** Pritesh P. Bhatt, Vrinda S. Thaker

**Affiliations:** Department of Biosciences, Saurashtra University, Rajkot, India

**Keywords:** *Cenchrus ciliaris*, chloroplast genome, phylogeny, Poaceae

## Abstract

*Cenchrus ciliaris* is an important pasture resource for arid region and owing to apomixis, it has been very important for development and distribution of cultivars and agro-types. In present study complete chloroplast genome of *C. ciliaris* was sequenced. The size of chloroplast genome is 138737 bp length with overall GC content 38.6%. It exhibited regular quadripartite structure with 81053 bp of LSC region, 16108 bp of SSC region and 20788 bp of each IR region. A total of 130 genes were identified, including 87 coding genes, 32 tRNAs, 7 ribosomal RNAs and 4 pseudogenes. Phylogenetic analysis was performed using 13 other members representing major subfamilies of Poaceae.

Buffel grass (*Cenchrus ciliaris*) is perennial, drought tolerant, deep rooted grass and responds more rapidly to rain. It is native to Africa, India and Indonesia. Once it is established it can withstand heavy grazing, has high nutritional value, spreads rapidly and therefore extremely valuable pasture resource for arid ecosystems like desert where very few resources are available (Janice 2005; Marshall et al. 2012). Facultative apomixis reported in *C. ciliaris* which is important for the development and distribution of cultivars and agro-types (Bray 1978). Because of these characteristics it is considered to be a ‘wonder crop’ (Hanselka 1988).

*C. ciliaris* was collected from Kutchh desert (23.85°N 69.72°E) and deposited at Department of Biosciences, Saurashtra University under voucher number SUPG005. Fresh leaves were kept for 48 hours in 4 °C to decrease starch level. Chloroplast DNA isolation was performed according to (Shi et al. 2012) and checked on 1% agarose gel electrophoresis. This DNA was sequenced using high throughput ion torrent genome machine with ion torrent server (torrent suite v3.2). Reference guided assembly was performed using CLC (mapping parameters: Mismatch cost =2, Insertion cost =3, Deletion cost =3, length fraction= 0.5, similarity =0.8) with already available chloroplast genome of *Cenchrus americanus* **(**NC_024171) as the reference genome. Contigs with more than 50 × sequence depth were used for reference guided assembly. The vote majority conflict resolution mode of CLC was used in order to ensure inclusion of only chloroplast specific reads thus avoiding contribution of nuclear and mitochondrial reads to the consensus sequence. Reads were also *de novo* assembled using CLC. Consensus sequence derived from reference assembly was compared and corrected with *de novo* assembly. Plastome annotation was performed in CpGAVAS (Liu et al. 2012) and DOGMA (Wyman et al. 2004). Further tRNA genes were confirmed using tRNAscan-SE 2.0 (Lowe and Eddy 1997). Complete chloroplast genome of *C. ciliaris* was submitted to NCBI GenBank (accession no. MH286942).

Chloroplast genome length of *C. ciliaris* is 138737 bp with overall 38.6% GC content. It exhibited regular quadripartite structure with 81053 bp of LSC region, 16108 bp of SSC region and 20788 bp of each IR region. A total of 130 genes were identified, including 87 coding genes, 32 tRNAs, 7 ribosomal RNAs and 4 pseudogenes. Total 17 genes including 8 protein coding genes, 6 transfer RNA genes and 3 ribosomal RNA genes present in IR region are found to be duplicated.

Phylogenetic analysis was performed using complete chloroplast genome of *C. ciliaris* with other 13 members of Poaceae. As *Cenchrus* belongs to Panicoideae 4 members selected from this group and from each subfamily of Poaceae one important member was selected. Phylogenetic tree clearly separates Panicoideae group containing 3 *Cenchrus* species, *Saccharum officinarum, Sorghum bicolor* and *Zea mays* from other members with nearly 100% bootstrap support (Figure 1). 

**Figure 1.**
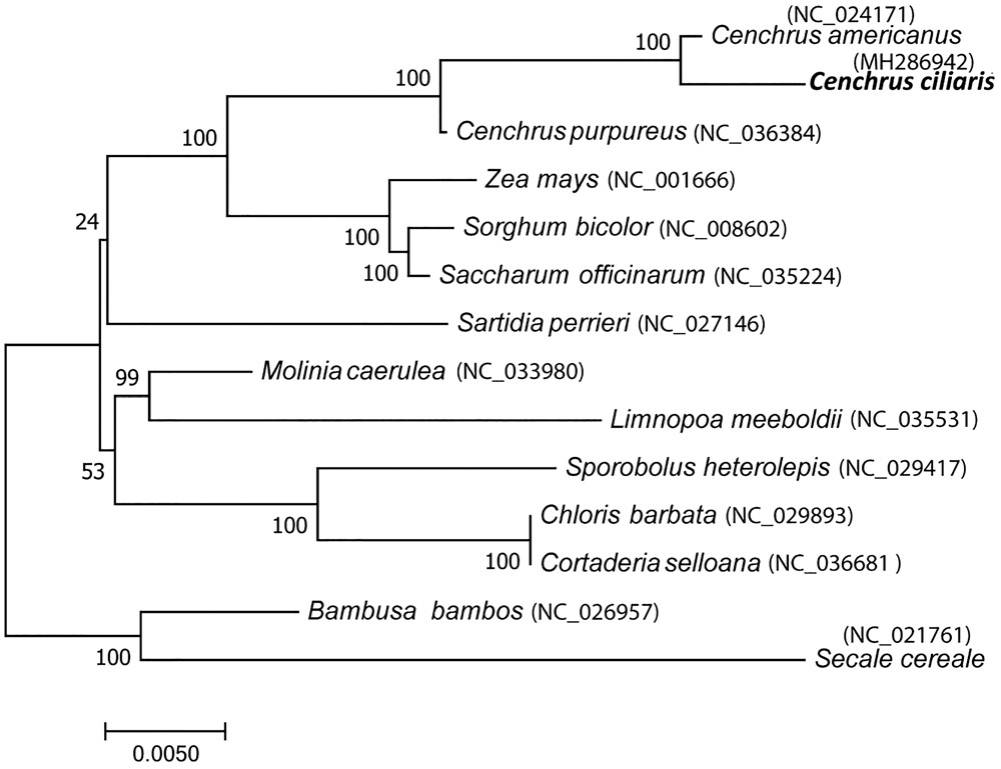
Phylogenetic tree based on complete chloroplast genome sequence of Cenchrus ciliaris and other 13 members of Poaceae. Phylogenetic tree constructed using maximum likelihood method and numbers on the each node are bootstrap values based on 1000 replicates.

As it is difficult to distinguish *C. ciliaris* from closely related other *Cenchrus* species and complete chloroplast genome provide effective tool for accurate identification and phylogenetic analysis.
